# Physiological and biochemical characterization of biochar-induced resistance against bacterial wilt of eggplants

**DOI:** 10.1098/rsos.230442

**Published:** 2023-08-09

**Authors:** Chaudhry Ali Ahmad, Muhammad Saleem Haider, Adnan Akhter

**Affiliations:** Faculty of Agricultural Sciences, Department of Plant Pathology, University of the Punjab, Quaid-e-Azam Campus, PO Box 54590, Lahore, Pakistan

**Keywords:** carbon sequestration, plant protection, *Ralstonia solanacearum*, soil amendment, organic agriculture

## Abstract

The abrupt variation in climatic patterns has become a global concern in terms of food security. Biochar, known to ameliorate climatic adversities by sequestering carbon and activating systemic resistance pathways in plants, has become increasingly relevant. Therefore, the study was aimed to characterize leaf waste biochar (LWB) by Fourier-transform infrared spectroscopy, scanning electron microscopy with energy dispersive X-ray (SEM-EDX) and X-ray diffraction analytical techniques as well as determination of its impact on the development of bacterial wilt (BW) in eggplant (*Solanum melongena*) caused by *Ralstonia solanacearum* (RS). The effect of LWB on the physiology and defence-associated biochemistry of eggplants was investigated thoroughly. Eggplants either inoculated (+RS) or uninoculated (-RS) were cultivated in potting mixture containing 3 and 6% (v/v) LWB separately. In comparison with substrate (soil only), percentage disease index was significantly reduced (71%) in plants grown in 6% LWB-amended treatments. Biochar-induced increase in level of total chlorophyll content as well as in biochemicals such as phenolics, flavonoids and peroxidases were evident on plants in terms of resistance response against BW. Moreover, biochar also significantly affected the level of NPK in the eggplants. In conclusion, biochar-triggered biochemical alterations played a pivotal role in the management of BW along with the curing of the disease-infested soils.

## 1. Introduction

Bacterial diseases are among the most harmful biological challenges that seriously deteriorate agricultural productivity [1,2]. Bacterial wilt (BW) caused by *Ralstonia solanacearum* is an economically significant disease affecting members of family Solanaceae in the tropical and subtropical regions, particularly if crop yield is geared toward the higher-priced off-season market [3]. Typically, the BW only affects one side of the plant or one mid-vein of the leaf [4]. Following infection, the entire plant soon wilts and perishes. BW has been reported to infect more than 300 species of 40 plus plant families. However, there is still no *R. solanacearum* management method developed to control the disease in eggplants in environment-friendly manner [5]. Furthermore, the widespread usage of chemical pesticides has highlighted numerous issues with plants, animals and human health. Therefore, researchers need to work on finding safer alternatives for the chemical pesticides [6–8].

Biochar, a charcoal-like substance created by the process of pyrolysis, which involves heating plant materials in an oxygen-limited atmosphere, has shown promise against a variety of plant diseases [9]. Biochar is a carbon-rich complex compound with characteristics derived from the pyrolysis conditions and type of initial raw material composition [10]. Although using biochar to improve soil quality has been done for centuries, researchers have just recently become particularly interested in it as a means of carbon sequestration and organic plant protection [11]. Application of biochar can change the way plants react to disease stress [12] in addition to having an impact on crop yield [13]. According to recent studies, using biochar and compost together has favourable effects on plant physiology and nutrient uptake [14].

Biochar, being an intriguing soil organic amendment, has shown the ability to inhibit the diseases of both bacterial and fungal origin either caused by soil- or air-borne pathogens. Previous studies reported the suppression of various pathogens infecting solanaceous host plants such as *Fusarium oxysporum* f. sp. *lycopersici* and *Alternaria solani* in tomato [14,15] and *Leveillula taurica* on pepper [16]. Biochar in soil activates plant's immune system in the form of acquired resistance systemic in nature and suppresses multiple pathogens (*Botrytis cinerea, Colletotrichum acutatum and Podosphaera aphanis*) of strawberry [17]. Gu *et al*. [18] reported the suppressive ability of pinewood biochar made at 700°C against *R. solanacearum* in tomato. While Chen *et al*. [19] used rice hull biochar against BW of tobacco crop.

Different organic raw material used for the biochar production are responsible for its diverse physico-chemical properties, thus resulting in variable response of the patho-system under investigation [14,15]. Therefore, it is equally important to evaluate different raw materials depending upon their local availability on the existing economic pathogens of the area. However, to date no study has been conducted on the impact of biochar on *R. solanacearum*-induced wilt in eggplants (*Solanum melongena*).

Eggplant (*S. melongena*) has annual global production of 54.1 million tons with 1.86 million hectares area of cultivation [20]. The production of eggplant suffers greatly from the numerous pests and diseases, including the emerging threat of BW [21]. *Ralstonia solanacearum* is problematic to crop production in tropical, subtropical and temperate regions. Overuse of agro-chemicals, in addition to causing environmental and health problems, also causes pathogenic strains to mutate, making them resistant to currently used disease management strategies [22]. Due to lethality, tenacity, extensive host range and widespread geographic distribution, *R. solanacearum* is recognized among the most significant phytopathogenic bacteria in today's world [23]. Subsequently, climate change also serves as an aid to the spread of several diseases resulting in reduced agricultural productivity.

The farming systems have become dependent on technology and chemical inputs to feed the growing global population (expected to be 9 billion in 2050) [24,25]. The plant pathogens are a major cause of deteriorating yield and quality of agricultural products [26]. Therefore, the aim of this study was to determine the ecological and economically viable strategy to increase agricultural yield by lowering the pathogen-induced losses, while conserving the biodiversity. The development of risk-free alternative disease management systems has received attention due to growing environmental contamination concerns caused by the pesticides. Consequently, it will be intriguing to assess the effects of biochar on the economically significant wilt-causing *R. solanacearum* on eggplants. Therefore, the purpose of this study was to characterize the locally produced biochar and determine its utility in preventing the establishment of BW on eggplants. The study also involves in-depth analysis of biochar-induced biochemical and physiological alterations stimulating plant growth and their impact on *R. solanacearum*–eggplant patho-system. According to the available literature, the present study was the first to elucidate the effect of biochar on the development of a soil-borne pathogen (*R. solanacearum*) in eggplants.

## 2. Material and methods

### 2.1. Isolation and molecular confirmation of *R. solanacearum*

During the eggplant growing season (February–March 2020–2021) survey was conducted (electronic supplementary material, figure S1) to collect the samples of eggplants infected with BW and to assess the disease prevalence (electronic supplementary material, table S1). The infected plant samples were collected from various localities of Punjab province of Pakistan as marked in map (electronic supplementary material, figure S1), along with their geographic coordinates as shown in electronic supplementary material, table S1. Stem slices of approximately 10 cm length were collected from the crown region of wilted plants. The segments were surface sterilized with 70% ethanol, and fragmented into small sections. The cut fragments were placed in a shaker containing double distilled sterilized water (5 ml) for 5 min at room temperature. Each bacterial suspension was streaked separately onto the bacterial growth medium, distributed evenly, and incubated at 28°C for 24 h [27].

Pure cultures were obtained by re-streaking a single colony from bacterial cultures isolated from stem on nutrient agar medium in sterilized environment. Bacterial cells were stirred in LB broth for 48 h at 37°C to prepare the inoculum. For inoculation, the final bacterial culture concentration was adjusted to 10^8^ colony-forming units ml^−1^ (CFU ml^−1^) (OD600 = 0.8) [28].

*Ralstonia solanacearum* identification based on colony morphology and polymerase chain reaction (PCR)-based molecular technique was carried out after 24 h of incubation [27]. The ‘CTAB technique’ was used to extract the complete genomic DNA from bacterial cells [29]. Universal primers 27F (GAGTTTGATCACTGGCTCAG) and 1492R (TACGGCTACCTTGTTACGACTT) for 16S RNA were used in the amplification reactions [30], which were carried out in 0.2 ml PCR tubes using a bio-red thermal cycler. The cyclers were set up to complete 30 cycles of 95°C for 60 s, 55°C for 50 s and 72°C for 60 s, followed by a final extension step of 5 min at 72°C. For sequencing, the PCR product was delivered to Macrogen Inc. (Seoul, South Korea). To determine the proportion of similarity with previously recognized pathogens, the acquired sequences were compared with the National Center for Biotechnology Information (NCBI) genome GenBank. The acquired sequences were analysed by the NCBI to determine its degree of similarity with previously reported pathogens.

### 2.2. Biochar production and physico-chemical characterization

*Syzygium cumini* leaves were pyrolysed at 450°C to produce the leaf waste biochar (LWB). The portable kiln technique known as top-lit updraft (TLUD) was used to make biochar with a few modifications [31]. Table 1 provides a description of the physico-chemical properties of soil, compost and LWB. Biochar produced was additionally characterized by the following methods:

**Table 1.** Characteristics of biochar made from leaf waste, compost and soil. CEC, cation exchange capacity; EC, electrical conductivity.

parameter	N (%)	P (%)	K (%)	C (%)	C/N ratio	Cu	Zn	Fe	CEC (mcq 100 g)	EC (mS cm)	organic matter (%)	pH
soil	0.06	2.09	1.84	1.09	15.24	0.22 ppm	1.08 ppm	1.59 ppm	122	0.59	0.599	7.99
LWB	0.92	0.64	0.724	45.69	48.59	0.11	0.022	0.52	12.92	1.71	59.92	9.37
compost	1.189	0.38	0.52	28.51	23.73	69 mg kg^−1^	459 mg kg^−1^	—–	—–	1.29	17.22	7.15

—– Parameters were not analysed.

#### 2.2.1. Scanning electron microscopy with energy dispersive X-ray analysis

Scanning electron microscopy with energy dispersive X-ray analysis (SEM-EDX) studies were carried out using a thermos Fischer Scientific USA FEI SEM Inspect S-50. Biochar powder was coated to aluminium struts using conductive carbon tape. SEM is operable at a vacuum of 2 × 10^−3^ Pa. Scanning electron micrographs were taken at 20 kV at magnifications of 500, 1000, 1500 and 2000 [32].

#### 2.2.2. Fourier-Transform infrared spectroscopy

Fourier-transform infrared (FTIR) spectra were used to assess the functional groups on the locally produced biochar surface. The biochar sample was placed on a germanium crystal in the attenuated total internal reflection mode for FTIR investigation. The spectra were recorded between 4000 and 800 cm^−1^ on a Bruker Vector 22 instrument [33].

#### 2.2.3. X-ray diffraction analysis

To investigate the material's crystallinity, X-ray diffraction (XRD) analysis was performed. Using the German-made X-ray diffractometer D8 advance from Bruker, biochar was subjected to XRD investigation. Cu K*α*1 radiation was used to take the XRD pattern, with a scan speed of 2.5° min^−1^ in the 2*θ* range of 10–80° [34].

### 2.3. *In vitro* impact of biochar on *Ralstonia solanacearum*

*Ralstonia solanacearum* was cultured in LB broth medium flasks for the *in vitro* evaluation to examine the impact of biochar on bacterial growth. In LB broth, biochar was introduced at 3% and 6% (v/v). At 37°C, a shaking incubator was used to incubate broth flasks. Using the Kim *et al*. [28] approach, the optical density of the bacterial culture was determined after 24 h of incubation (10^8^ CFU ml^−1^ at OD600 = 0.8) [28]. Five days' growth readings were taken. This is how percentage growth inhibition (PGI) was calculated [35]

$$\mathrm{PGI}\;\text{\%}=\frac{\mathrm{control}-\;\mathrm{treatment}}{\mathrm{control}}\;\times 100.$$

### 2.4. Plant material and experiment set-up

Surface sterilization of eggplant seeds was performed by using 3.8% NaOCl solution (50% commercial bleach; provided by Ittehad Chemicals, Empress Road, Lahore) for 10 min followed by washing three times with autoclaved distilled water [36]. On the basis of prevalence among all of the sampling locations, out of the two isolates (Isolate-01 and 02) of *R. solanacearum*, Isolate-01 from Faisalabad (31″32′42″ N, 73″49′35″ E) was selected for further bioassays as it has shown maximum disease incidence and severe observable symptoms, while Isolate-02 was only found at Toba Tek Singh (30″57′15″ N, 72″28′03″ E) (electronic supplementary material, table S1) along with Isolate-01, as revealed by sequence analysis. Prior to transplantation, the seeds were sown in autoclaved soil pots and kept at 24°C in the glasshouse for four weeks. The seedling's roots were soaked in bacterial culture after artificial wounding for up to 3 min prior to planting in pots. After one week of seedling transfer, the rhizospheric soil region was drenched with 50 ml of bacterial culture (10^8^ CFU ml^−1^) [37].

Different volumetric concentrations (3 and 6%) of biochar were used as soil amendments either inoculated or uninoculated with *R. solanacearum* (Isolate-01) in the experimental set-up. The bulk density of the formalin-sterilized soil used as basic potting material was 1.30 g cm^−3^ (Pakistan Council of Research in Water Resources (PCRWR)). The sandy loam soil had composition of 5.6% clay (2 mm), 42.9% silt (greater than 2 mm) and 52.2% sand (greater than 63 mm) particles [14]. There were five replicates assigned to each treatment in the completely randomized (CRD) experiment design with two experimental repeats.

### 2.5. Eggplant growth parameter assessment

The plants were harvested 40 days after inoculation and processed to measure the agronomic plant physiological characteristics such as plant height, root length, above and below ground biomass dry weights. Eggplant roots and shoots were chopped apart and dried in an oven at 70°C until they reached a steady weight [38]. For Nitrogen (N), phosphorus (P) and potassium (K) assessment in the eggplant shoots Kjeldhal method was employed to analyse [39]. For analysis, the dried plants were ground into a powder. To achieve the best results, potassium sulfate and copper sulfate were used as catalysts in a 9 : 1 ratio and 0.5 g of the sample was digested with 10 ml H_2_SO_4_ at 420°C for 2 h. Total P and K in plant material were measured by the International Center for Agricultural Research in the Dry Areas (ICARDA) manual wet-digestion technique [39]. The concentrations of P and K were measured using spectrophotometers and flame photometers, respectively [39].

### 2.6. Disease assessment

Slightly modified method proposed by Winstead and Kelman was adopted to record the disease ratings [40]. The disease rating scale ranges between 0 and 5, with 0 corresponding to immune (I), 1 for highly resistant (HR), 2 for resistant (R), 3 for moderately resistant (MR), while 4 and 5 represent susceptible (S) and highly susceptible (HS) plant response, respectively.

Disease prevalence and index was evaluated based on the basis of following formula [41]:

$$\mathrm{per}\;\mathrm{cent}\;\mathrm{disease}\;\mathrm{index}\;(\mathrm{PDI})\;=\frac{\mathrm{sum}\;\mathrm{of}\;\mathrm{all}\;\mathrm{rating}\times 100}{\mathrm{total}\;\mathrm{no}.\;\mathrm{of}\;\mathrm{observations}\times \mathrm{maximum}\;\mathrm{rating}\;\mathrm{grade}}.$$

Forty days after inoculation, disease incidence of BW was calculated as percentage of infected plants in treatment [42,43],

$$\mathrm{disease}\;\mathrm{incidence}\;(\text{\%})\;=\frac{\mathrm{number}\;\mathrm{of}\;\mathrm{diseased}\;\mathrm{plants}}{\mathrm{total}\;\mathrm{number}\;\mathrm{of}\;\mathrm{plants}}\times 100.$$

The percentage of protection provided by biochar was calculated by the formula given below, where X represents the PDI of untreated control plants and Y represents the PDI of treated eggplants [44].

$$\mathrm{protection}\;(\text{\%})\;=\frac{X-Y}{X}\times 100.$$

### 2.7. Biochemical analysis of different resistance indicators in eggplant in response to biochar

The amounts of soluble proteins were calculated by taking 5 ml of alkaline reagent (50 ml of 2% Na_2_CO_3_ prepared in 0.1 N NaOH and 1 ml of 0.5% CuSO_4_ prepared in 1% potassium sodium tartrate) and 0.5 ml of Folin's reagent (diluted by 1 : 3 v/v) were combined with 1 ml of plant extract. The colour change was recorded after 30 min at the 750 nm wavelength [45].

The total flavonoid content of crude extract was determined by the aluminium chloride (AlCl_3_) colorimetric method described in [46]. While the method given in [47] was used to measure the activity of catalases.

In order to determine the photosynthetic pigment contents (chlorophyll), fresh 0.5 g leaf tissue was mashed in acetone (80%) using a pestle and mortar. To determine the amount of chlorophyll a, chlorophyll b and carotenoid content, the filtrate was centrifuged for 5 min at 10 000*g*. The filtrate's absorbance was then measured at 470, 652 and 665 nm [48,49]. Peroxidase activity was analysed by taking enzyme extract (0.5 ml) in reaction mixture (2 ml of phosphate buffer (0.1 mol l^−1^; pH 6.8), 1 ml of pyrogallol and 1 ml of 0.05 mol l^−1^ H_2_O_2_ (5 : 5 in H_2_O_2_ and distilled water)) followed by an incubation at 25°C. The reaction was ceased by the addition of H_2_SO_4_ (2.5 mol l^−1^). The reading was taken at wavelength of 420 nm against a pyrogallol-removed blank.

Using the modified Folin–Ciocalteu technique, the total phenolic content was calculated [50]. Na_2_CO_3_ and the Folin–Ciocalteu reagent (0.5 ml) were combined with the extracts (1 ml). After 10 min of incubation, the absorbance value was measured at 760 nm using a spectrophotometer (Perkin Elmer Lambda-EZ200). Gallic acid equivalent (GAE) was used to express the overall status of phenolic compounds in samples.

### 2.8. Statistical analysis

The data analysis was performed with the Statistics 8.1 software. The data were analysed for homogeneity of variance. Afterwards the treatments were subjected to two-way analysis of variance (ANOVA) and the factors of analysis were soil composition and *R. solanacearum*. All the means were separated with the Tukey's honestly significant difference (HSD) all-pairwise comparisons test (*p* ≤ 0.05).

## 3. Results

### 3.1. Molecular confirmation of *R. solanacearum*

**Figure 1.**
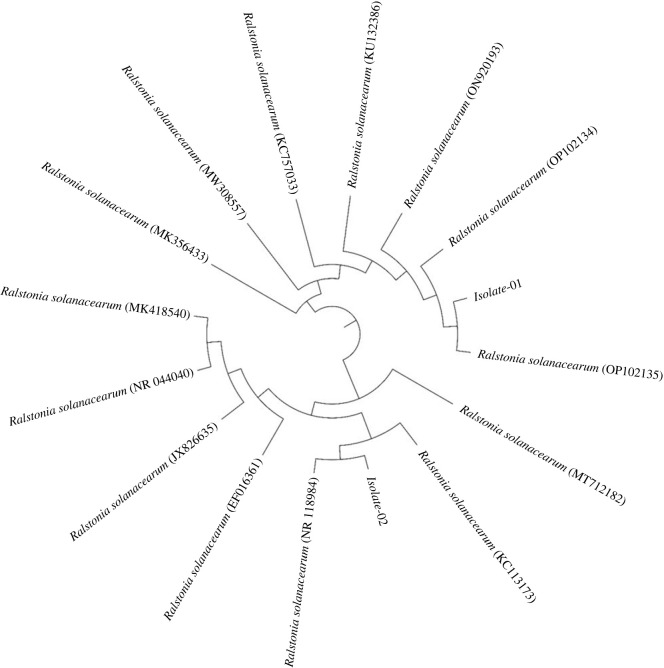
Phylogenetic tree of *Ralstonia solanacearum* for isolates showing close homology (100%) with the NR118984 and OP102135.1 isolates, respectively.

The PCR amplifications size of approximately 1400 bp were obtained and subjected to further sequence analysis. The PCR products sequences were submitted to the Genbank and received accession numbers for Isolate-01 and 02 (OQ587616 and OQ587617, respectively) for 16S ribosomal RNA. BLASTn comparison analysis of the 16S Isolate-01 (GenBank accession no. OQ587617) has similarity (100%) with *R. solanacearum* isolates ‘OP102135 and OP102134′ reported in Chile from hosts *Solanum lycopersicum* and *Cucumis sativus*, respectively. While Isolate-02 (GenBank accession no. OQ587616) has shown close homology (100%) with the *R. solanacearum* from Australia (NR118984), host/source unknown, as well as shown similarity with *R. solanacearum* isolated from dust (accession number KC113173) from Iran (figure 1).

### 3.2. Chemical and physical assessment of biochar

**Figure 2.**
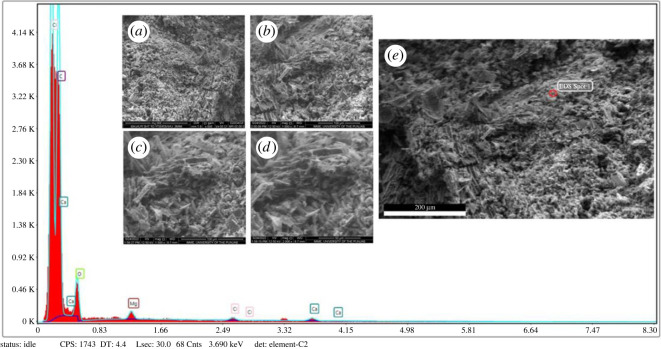
(*a–d*) SEM images taken at 500, 1000, 1500 and 2000 magnifications representing the pelletized structure, channels and pore size of leaf waste biochar prepared at 450°C; (*e*) EDX elemental analysis of leaf waste biochar showing the presence of carbon, magnesium, chlorine, oxygen and calcium.

The magnifications of 500, 1000, 1500 and 2000 were employed for scanning electron microscopy micrographs. The SEM image at 500 magnifications showed a fragmented pelletized structure of biochar, whereas the image at 2000 magnification showed tubular structures. The micrograph taken at 1500 magnification also revealed several tubular pores of varying sizes that were attached to the walls of particles (figure 2*a–e*).

The elemental or SEM-EDX analysis of LWB demonstrated a high concentration of minerals. The C contents at EDS spot were the maximum (70.15) (figure 2). In addition to that, magnesium, chlorine, oxygen and calcium had produced the values of 1.8, 1.19, 24.45 and 2.41, respectively (figure 2*e*).

**Figure 3.**
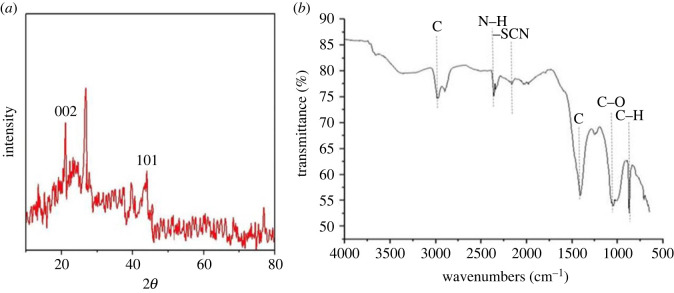
(*a*) X-ray diffractogram of biochar indicating peaks symbolizing graphite structures at 101 and 002, and, (*b*) FTIR spectrum of leaf waste biochar prepared at 450°C highlighting carbon-related compound peaks, along with the presence of amine group, thiocyanate (–SCN) functional group and epoxy and oxirane rings.

X-ray diffraction analysis using Bragg's angle of 2*ϴ* revealed many unidentified peaks characterized as unidentified organic substances, along with heterogeneous surface of LWB. However, the region between 20° and 30° highlighted the graphite layers (figure 3*a*). The peak at 21° indicates the usual crystalline structure of cellulose, whereas the peak at 44° also symbolizes graphite (101). Graphite 002 is represented by peak at the 26°. The expanded region comprised aromatic layers with perpendicular structures of crystalline nature.

The FTIR spectrum of biochar showed variance in band spectra at 2986.78 cm^−1^ wavenumber with a carbon-related compound peak as well as a visible peak at 2360.59 cm^−1^ suggesting N–H bending belonging to the amine group. Thiocyanate (–SCN) functional group represented by the peak at 2161.40 cm^−1^ wavenumber. The C≡C bending of LWB indicated by the spectral peak at 1051.13 cm^−1^. The spectral peak at 872.76 cm^−1^ and 1250 cm^−1^ wavenumbers revealed the existence of epoxy and oxirane rings (figure 3*b*).

### 3.3. *In vitro* impact of biochar on *R. solanacearum*

**Figure 4.**
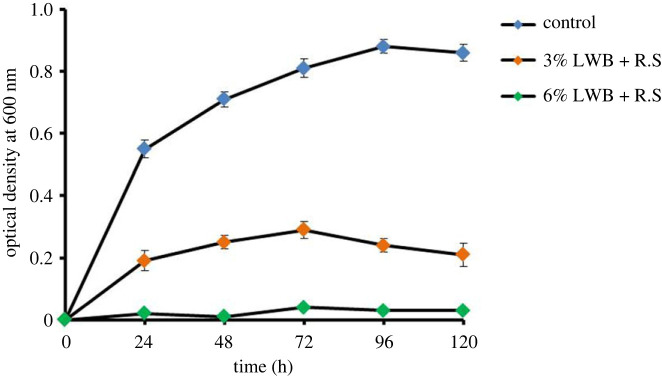
Effects of leaf waste biochar (LWB) *in vitro* on *Ralstonia solanacearum* (R.S). A spectrophotometer (600 nm wavelength) was used to determine the optical density of the bacterial culture. All values represent mean ± s.e., (*n* = 3).

In order to examine the *in vitro* impact of biochar on *R. solanacearum* (Isolate-01) growth, LB broth medium were altered with 3% and 6% of biochar concentration (v/v). When 6% LWB was applied, bacterial growth was greatly decreased (85.71 and 95.40%) compared with 3% LWB and the unaltered control, respectively (figure 4).

### 3.4. Impact of biochar and *R. solanacearum* on eggplant growth

*Ralstonia solanacearum* (Isolate-01) and soil substrate composition had a significant impact on the evaluated plant growth parameters of eggplant. However, both above- and below-ground plant biomass production as well as root and shoot length were strongly influenced (*p* ≤ 0.001) by the interaction between soil composition and *R. solanacearum* (table 2). The pathogen inoculation generally had an inhibitory effect on plant growth parameters.

**Table 2.** The level of significance of the relationship between *Ralstonia solanacearum* (RS) and soil substrate composition (SC) on eggplant development and physiological parameters was determined by the results of a two-way ANOVA.

treatments	shoot length	shoot dry weight	root length	root dry weight	N	P	K	catalase	flavonoids	phenol contents	soluble proteins	peroxidase	chlorophyll contents	carotenoids
SC	***	***	***	***	***	***	***	***	***	***	Ns	***	***	***
RS	***	***	***	***	***	***	***	***	***	***	***	***	***	***
SC × RS	***	**	Ns	**	**	***	*	*	Ns	Ns	Ns	***	**	Ns

*** *p* ≤ 0.001, ** *p* ≤ 0.01, *p ≤ 0.05 and Ns, non-significant. The data analysis represents five replicates for each parameter.

Plant height was significantly reduced in all *R. solanacearum* inoculated treatments (table 2). However, the highest plant shoot length (30.5 cm) was observed in the uninoculated (−RS), 6% LWB soil amendment, which was 41% higher than the soil with no biochar. Root length has shown highly significant effect (*p* ≤ 0.001) of factors soil substrate composition and *R. solanacearum*. The highest root length (14.8 cm) was recorded in the uninoculated (−RS) 6% LWB soil-amended treatment. While, the presence of BW significantly reduced the dry weights of plant parts in all treatments (table 3). The highest shoot dry weight (3.43 and 3.3 g) was recorded in 6% LWB-amended soil treatment, both in the absence and presence of *R. solanacearum*, respectively. The root dry weight was significantly higher (67.6%) in inoculated 6% LWB soil amendment in comparison with the plants grown in unamended soil substrate.

**Table 3.** Effect of *Ralstonia Solanacearum* (RS) on eggplants grown in different soil substrate compositions comprising soil only, 3% and 6% leaf waste biochar. All values represent mean ± s.e., (*n* = 5). Mean values with different lettering suggesting significant difference as per Tukey's HSD test (*p* ≤ 0.05).

treatments	root length (cm)	shoot length (cm)	root dry weight (g)	shoot dry weight (g)	nitrogen (N) (%)	phosphorus (P) (ppm)	potassium (K) (ppm)
S − RS	10.3 ± 0.27d	21.3 ± 0.84d	0.64 ± 0.02d	2.272 ± 0.16c	1.74 ± 0.08c	0.28 ± 0.01c	1.61 ± 0.04b
S + RS	8.18 ± 0.28e	17.1 ± 0.42e	0.52 ± 0.03e	1.582 ± 0.31d	1.216 ± 0.01d	0.12 ± 0.01d	1.35 ± 0.01c
S + 3% LWB − RS	12.52 ± 0.3c	28.05 ± 0.67b	0.77 ± 0.03c	2.7 ± 0.19b	2.16 ± 0.02b	0.53 ± 0.01b	1.69 ± 0.1b
S + 3% LWB + RS	10.84 ± 0.26d	25.55 ± 1.07c	0.68 ± 0.01d	2.308 ± 0.12bc	1.83 ± 0.073c	0.27 ± 0.05c	1.41 ± 0.01c
S + 6% LWB − RS	14.8 ± 0.57a	30.05 ± 0.64a	1.08 ± 0.06a	3.414 ± 0.27a	2.5 ± 0.15a	0.67 ± 0.05a	2.02 ± 0.06a
S + 6% LWB + RS	13.34 ± 0.22b	29.15 ± 0.52ab	0.86 ± 0.04b	3.3292 ± 0.17a	2.4 ± 0.12ab	0.56 ± 0.02b	1.91 ± 0.09a

Data represents mean ± s.d. (*n* = 5). Mean values with different lettering suggesting significant difference within each column as per Tukey's HSD test (*p* ≤ 0.05).

As far as N, P and K contents of eggplants are concerned, there was significant interactive effect between ‘SC × RS’ on eggplant nutrient profile comprising nitrogen (N), phosphorus (P) and potassium (K) contents (table 2). The 6% LWB soil treatment had the highest N contents of 2.5%, while no significant reduction in N contents was recorded in the presence of *R. solanacearum* in LWB containing soil (table 3). Similarly, plants raised in 6% LWB-amended soil had the highest value of P and K contents as well. While, the level of P content in the remaining biochar-amended treatments inoculated with *R. solanacearum* was significantly reduced. The minimum (0.12 ppm) level of P contents was found in *R. solanacearum*-infected plants grown in soil control. *Ralstonia solanacearum*-inoculated plants grown in 6% LWB + RS has 42.88% higher K contents when compared with the soil only treatment.

### 3.5. Influence of biochar on disease development

The response of the eggplant to *R. solanacearum* (Isolate-01) inoculation ranged from resistant to highly susceptible with respect to soil composition (table 4). Plants cultivated in soil (S + RS) treatment showed a highly susceptible response to BW, with the highest PDI of 84% and 100% disease incidence. Although, the biochar-amended soil substrate had an inhibitory effect on the growth of pathogen. Although there was a noticeable decrease in PDI, DS and DI among the plants grown in biochar, the highest plant defence against the development of BW was observed in the treatment that contained 6% biochar (S + 6% LWB + RS), with lowest (12%) PDI, DS (9.41%) and DI (20%). In comparison with soil only treatment, 6% LWB induced 86% protection against BW on the eggplants while it was 48% in case of plants grown in 3% LWB (table 4).

**Table 4.** Estimation of disease incidence (DI), disease severity (DS), per cent disease index (PDI), percentage protection, plant disease response (PDR) from highly susceptible (HS) to susceptible (S) and resistant (R) response based on disease ratings for bacterial wilt in eggplants cultivated in different soil substrate compositions.

treatments	DI (%)	DS (%)	PDI	% protection*	PDR	disease rating scale
S + RS	100	66.71a	87	–	HS	5
S + 3% LWB + RS	60	37.59ab	47	48	S	4
S + 6% LWB + RS	20	9.41b	16	86	R	2

*Plant protection from *Ralstonia solanacearum* of biochar amended treatments in comparison with soil only treatment.

### 3.6. Biochemical analysis of resistance indicators activation in eggplants in response to biochar

**Figure 5.**
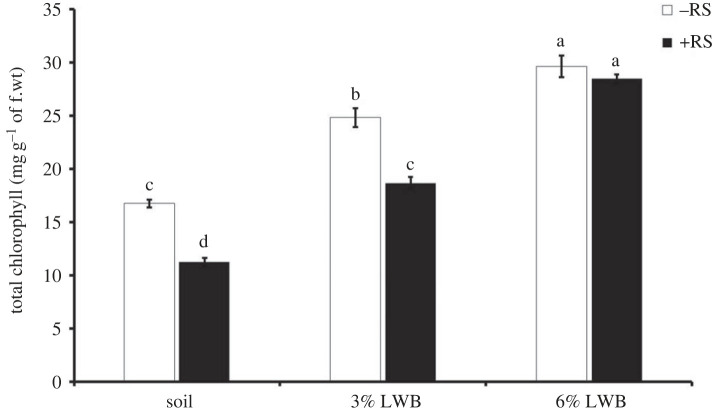
Effect of leaf waste biochar and *Ralstonia solanacearum* on total chlorophyll content of eggplant cultivated in following soil compositions; soil, soil with 3% LWB, and soil with 6% LWB, either inoculated (+RS) or uninoculated (−RS). All values represent mean ± s.e., (*n* = 5). Bars with different top lettering suggesting significant difference as per Tukey's HSD test (*p* ≤ 0.05). f.wt, fresh weight.

Plant chlorophyll contents varied significantly with the interactive effect of RS × SC (*p* < 0.05) (table 2). The soil substrates containing biochar induced significant improvement in the chlorophyll concentration of eggplants (figure 5). Highest chlorophyll contents 29.65 ± 1.02 and 28.5 ± 0.4 were observed in plants grown in ‘soil + 6% LWB’ either uninoculated (−RS) or inoculated (+RS), respectively. Lowest chlorophyll contents were observed in soil only potting medium inoculated with *R. solanacearum* which were 60.20% lower than the ‘soil + 3% LWB + RS’ treatment.

However, inoculated plants with 6% LWB demonstrated minimal inhibitory effect on chlorophyll contents.

**Figure 6.**
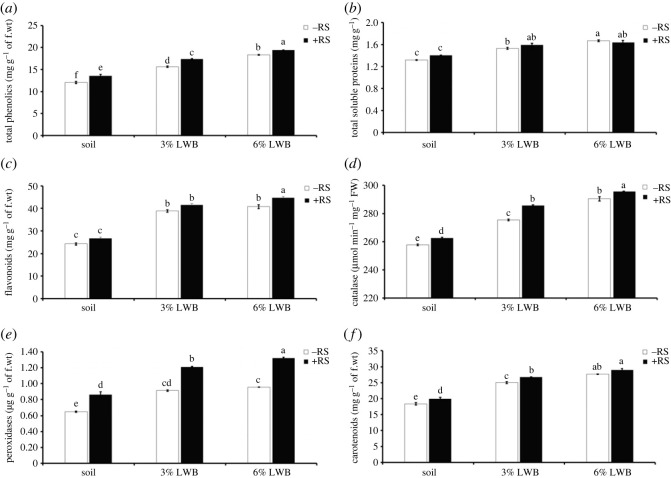
Effect of leaf waste biochar and *Ralstonia solanacearum* on total phenolics (*a*), total soluble proteins (*b*), flavonoids (*c*), catalases (*d*), peroxidases (*e*) and carotenoids (*f*) of eggplant cultivated in following soil compositions; soil, soil with 3% LWB, and soil with 6% LWB, either inoculated (+RS) or uninoculated (−RS). All values represent mean ± s.e., (*n* = 5). Bars with different top lettering suggesting significant difference as per Tukey's HSD test (*p* ≤ 0.05).

Total phenolics of eggplant were significantly (*p* ≤ 0.001) altered by the BW pathogen and soil substrate composition. The rise of total phenolics was found directly related to the increase in biochar concentration (figure 6*a*). The concentration of total phenolics in the ‘soil + 3% LWB’ with or without the pathogen was 28.11 and 29.64% higher than their respective controls. While in case of plants grown in 6% LWB, an increase of 11.93 and 17.46% was recorded in comparison with the respective treatments received 3% biochar amendment. On the contrary, total soluble proteins were significantly (*p* ≤ 0.001) influenced by the BW (figure 6*b*). The lowest protein contents of 1.32 mg g^−1^ of fresh weight were recorded for uninoculated plants raised in potting mixture without any biochar, while the highest (1.67 mg g^−1^ of fresh weight) were in 6% LWB amended substrate. However, in both the pathogen inoculated and uninoculated 3% LWB amended soil composition, total soluble protein contents of 1.53 and 1.59 mg g^−1^ of fresh weight were calculated.

For plants grown in 6% LWB, either inoculated (+RS) or uninoculated (–RS), the highest flavonoids concentrations were highest (44.68 and 40.76 mg g^−1^ of fresh tissue, respectively) (figure 6*c*). In the absence of biochar, flavonoids' concentrations were found to be lowest (24.18 mg g^−1^ of fresh tissue) in soil only treatment. All of the studied variables had a significant (*p* < 0.05) interactive impact on how much catalase was produced. The catalase activity in eggplants was, however, significantly influenced by an interaction between the soil substrate and *R. solanacearum*. In biochar-altered treatments, eggplants grown in 6% LWB-inoculated (+RS) treatments showed the highest catalase activity (295.62 µmol min^−1^ mg^−1^ of fresh weight), representing an increase of 2.26% over their healthy counterpart (figure 6*d*). By contrast, soil only treatment without pathogens showed the lowest catalase activity (257.006 µmol min^−1^ mg^−1^ of fresh weight). However, there was no discernible difference between the 3 and 6% LWB amended treatments, whether or not it had been infected with *R. solanacearum*. The pathogen-inoculated treatment's catalase activity was ranked from highest to lowest in the following order: 6% LWB > 3% LWB > S.

The level of peroxidase in the disease-stressed eggplant foliar region was significantly affected by the addition of biochar (table 2) among the +RS plants grown in 3 and 6% LWB, the maximal peroxidase activity was calculated to be 1.21 and 1.32 µg g^−1^ of fresh weight, respectively (figure 6*e*). However, in 6% LWB inoculated (+RS) treatment an increase of 9.09% over 3% LWB + RS was calculated. While, both of the stated variables (soil substrate and *R. solanacearum*) had a highly significant (*p* ≤ 0.001) influence on the activity of carotenoids in eggplants. Highest carotenoids (28.83 mg g^−1^ of fresh weight) contents were recorded in eggplants raised in 6% LWB under pathogen stress. Comparing the carotenoids content of the 6% biochar amended treatments (+RS) with the 3% LWB and soil only treatments under pathogen stress, there was a difference in percentage (2.15 and 8.94%), respectively (figure 6).

## 4. Discussion

Biochar application enhanced the porosity and infiltration rates, while lowering the bulk density and improving the granular structure of soil [51]. Our analysis of biochar produced from leaf waste, at a magnification of 2000×, of scanning electron microscope revealed the highly porous structure and the presence of distinct tubular canals (figure 2). The production of crops has been seriously hampered by the soil degradation brought on by intensive agriculture. Poor or compacted soil structure increases the risk of disease, as the most significant indication of soil quality is soil porosity [52]. Xu *et al*. [53] demonstrated that SEM-EDX analysis of microscopic surface features of biochar modified the soil elemental complex composition, as the biochars possess the tendency of adsorption of minerals, thus modifying the soil environment considerably. In addition to that, high carbon contents of LWB plausibly supported the beneficial microbial activity in the soil. The diversity and number of microorganisms in the soil, increase in organic carbon, and the adaptable nature of biochar are potential mechanisms behind improvement in soil characteristics [54]. Typically focusing on the soil, then pores and particle size of biochar are major contributors in the soil health improvement [55].

Similarly, our results revealed that the LWB had a good amount of mineral nutrients, according to SEM-EDX examination. The analysis made it clear that the macro- (K, P) and micro-nutrient (Ca, S, Cl) contents of the biochar may be associated with its phyto-stimulating properties (figure 1). Previously, it has been demonstrated that biochar with a high Ca concentration can enhance the soil's exchangeable cation status, supporting the theory that the exchangeable cations in degraded soils were originally supplied by the biochar [7]. The heterogeneity of the biochar material due to the mineral compositions has been substantiated by the XRD examination carried out in this experiment (figure 3*a*). Sharp peaks between 20 and 30° depict the graphite carbon substance found in biochar. In the past, Shaaban *et al*. [56] found that the presence of cellulose components caused the biochar to have strong peaks at various angles when produced at low pyrolysis temperature.

We analysed the biochar predominantly made up of biopolymer residues, such as lignin and cellulose, after pyrolysis temperature is raised to 450°C or above. The aromatic structure of the biochar was confirmed by spectral peaks from the FTIR that showed different bonding in carbon with other elements (figure 3*b*). Previous reports in line with our findings suggested that biochar formed at temperatures exceeding 480°C has high porosity, aromatic carbon compounds as well as an amorphous structure [7].

Our findings also indicated that treatments with higher LWB concentrations exhibited increased eggplants agronomic and physiological growth characteristics, which could be due to their enhanced capacity to reduce soil pH. The liming effect of the biochar may also have contributed to the overall boost in growth as well as improving the plant's ability to use nutrients [57].

The direct toxicity of biochar to soil-borne pathogens may be the cause of the control of disease-causing microbes; although, in leaf infections, the likely interaction still needs to be clarified. Our findings suggested that eggplant grown in potting soil that has been biochar-amended is more protected against *R. solanacearum*. A unique habitat with high carbon content, organic acids and small amounts of phenols that might trigger the hormesis response with the addition of biochar to the soil substrate [57].

The ability of biochar made from various feedstocks to inhibit infection varies. While the effect of biochar in terms of disease resistance is determined at the host and pathogen interface as biochar-borne chemicals induce systemic resistance within the plants to cope with the infectious agents [15]. According to Jaiswal *et al*. [58], eucalyptus-based biochar is more effective than biochar made from greenhouse waste in suppressing the *Rhizoctonia solani* infection of cucumbers. Moreover, mixing biochar with compost has the added benefit of reducing disease occurrence and boosting plant growth and output. According to Akhter *et al*. [15], biochar and compost application suppressed *Fusarium oxysporum* f. sp. *lycopersici* in tomatoes due to synergic effect, but Debode *et al*. [59] showed little or negligible effect of biochar-amended compost on lettuce basal rot (*R. solani*). Such a variable response of patho-systems to the biochar application makes it very essential to assess different biochars based on feedstock against different hosts and pathogens.

To achieve the intended results, whether for improving plant health or suppressing disease, different biochars with varying compositions do not adhere to the uniform application rate principle [58,60]. The incidence, severity, vegetative as well as physiological responses of the plants to the BW disease were also affected by the rate of biochar application. Similarly, in our work, 6% LWB was more effective than 3% LWB in reducing the establishment of *R. solanacearum* in eggplants (table 4). Increased disease suppression at higher doses of biochar is attributed to enhanced adsorption of root exudates thus reducing the motility of *R. solanacearum* [18]. Elad *et al*. [9] discovered an effective management of *Botrytis cinerea* at higher biochar application, while, Zwart *et al*. [61] found suppression in stem lesions induced by *Phytophthora* spp*.* in ornamental tree species (*Acer rubrum* and *Quercus rubra*) cultivated in 5% biochar-amended potting media produced from raw material of pine (*Pinus* spp.) origin. Contrary to our findings, Harel *et al*. [17] showed that smaller concentrations of biochar reduced disease inhibition of foliar pathogens that cause powdery mildew and grey mould on strawberries. Therefore, the type and concentration of biochar in the potting media strongly influences the ability to effectively suppress diseases and promote plant growth.

The chlorophyll contents were significantly increased in eggplants grown in higher doses of biochar both with and without disease stress (figure 5). The increase in chlorophyll content was mainly attributed to enhanced uptake of nutrients especially of N and Mg [62], because of biochar-induced increase in pH of soil and reduced nutrient leaching [63]. Our results had shown that the antioxidants (phenolics, catalases, flavonoids and etc.) might have played their role in protection of photosynthetic machinery and provided defence against wilt-induced stress [64].

Eggplants cultivated in soil supplemented with leaf waste biochar had higher levels of defence-associated biochemicals. We found the higher levels of defence-associated metabolites such as phenolics, catalases and peroxidases, along with enhanced growth and disease protection in the *R. solanacearum*-inoculated plants grown in 6% biochar (figure 6). Phenolics work by increasing the defence proteins that eventually prevent the pathogen spread by adding structural hindrances such as lignifying the cell wall and also by reducing the stress induced by reactive oxygen species [65,66]. Previously, increased production of peroxidase was reported to promote the lignification in tobacco in response to resistance inducers against BW [67]. The production of phenolics and antioxidants in tomato has been linked with the boosting of plant defence against early blight [43]. According to our findings, *R. solanacearum* attack on eggplants in the biochar-based soil amendment considerably influenced the levels of catalase and peroxidase synthesis, which in turn helped to mitigate the stress by bacterial infection [44].

Findings of the present study greatly advance our knowledge of how biochar made from *S. cumini* leaves contributes to the development of BW resistance in eggplants and the recycling of waste products into carbon-rich soil amendments. Organic amendments plausibly increased the soil's organic carbon contents and eventually rendered the soil structure permeable, and consequently facilitated roots development deeper into the soil to gather more nutrients. These results may also be exploited to develop organic materials that can be used as substitute disease management methods for controlling BW in eggplants. Also, because biochar is more stable in the soil, it offers longer-lasting plant protection.

## 5. Conclusion

Here, we demonstrated that the higher LWB concentration influenced eggplant's response to the invasive *R. solanacearum*. Organic soil additions based on biochar have the ability to boost plant defence and control newly developing biotic challenges to eggplant production. The raw material used for pyrolysis affects the physico-chemical characteristics of biochar, which in turn determine how plants will respond (biochemically and physiologically) to phyto-pathogens. Researchers ought to examine biochars of different origin against economically important pathogens that affect the same crop, because doing so will undoubtedly increase the biochar's usefulness and market worth.

Understanding the mechanisms of action of biochar against both soil-borne and foliar pathogens is expected to advance in the upcoming years. We think that biochar's effects on the rhizosphere microbiota and plants' natural defence may contribute to the improved plant development. In the recent future, we expect there to be increased interest in biochar from the perspective of the plant protection sector and a suitable waste management strategy. Future studies should focus on the in-depth analysis of biochar derived from various wastes to understand the interactive mechanisms between biochar, hosts and their pathogens. This will not only help in development of effective disease management strategies but also in formulating suitable biochar-based commercial products.

## Ethics

This work did not require ethical approval from a human subject or animal welfare committee.

## Data Availability

The datasets supporting this article have been uploaded as part of the electronic supplementary material. The genomic data generated have been deposited in GenBank (accession nos.: OQ587616 and OQ587617). The electronic supplementary material contains information about survey of eggplant growing areas along with geological coordinates (google earth) and bacterial wilt disease incidence data (represented as electronic supplementary material, table S1) and electronic supplementary material, table S2 showing elements present on EDS Spot [68].
